# Programmed Cell Death Ligand (PD-L)-1 Contributes to the Regulation of CD4^+^ T Effector and Regulatory T Cells in Cutaneous Leishmaniasis

**DOI:** 10.3389/fimmu.2020.574491

**Published:** 2020-10-22

**Authors:** Rafael de Freitas e Silva, Rosa Isela Gálvez, Valéria Rego Alves Pereira, Maria Edileuza Felinto de Brito, Siew Ling Choy, Hannelore Lotter, Lidia Bosurgi, Thomas Jacobs

**Affiliations:** ^1^Protozoa Immunology, Bernhard Nocht Institute for Tropical Medicine, Hamburg, Germany; ^2^Department of Natural Sciences, University of Pernambuco, Garanhuns, Brazil; ^3^Department of Immunology, Aggeu Magalhães Institute—Oswaldo Cruz Foundation, Recife, Brazil; ^4^Department of Molecular Parasitology and Immunology, Bernhard Nocht Institute for Tropical Medicine, Hamburg, Germany; ^5^I. Department of Medicine, University Medical Center Hamburg-Eppendorf, Hamburg, Germany

**Keywords:** leishmaniasis, CD4 T cell, co-inhibitory receptors, PD-1 (CD279), PD-L1 (B7-H1 CD274)

## Abstract

Cutaneous Leishmaniasis (CL) affects up to one million people every year and treatments are costly and toxic. The regulation of the host immune response is complex and the knowledge of how CD4^+^ T cells are activated and maintained during *Leishmania* infection is still limited. Current therapies aim to target programmed cell death (PD)-1 and programmed cell death ligand (PD-L)-1 in order to boost T cell activity. However, the role of the PD-1/PD-L1 axis during *Leishmania* infection is still unclear. In this study, we found that patients with active and post-treatment CL displayed different subsets of CD4^+^PD-1^+^ T cells. Accordingly, *L. major-*infected mice upregulated PD-1 on activated CD4^+^ T effector cells and PD-L1 on resident macrophages and infiltrating monocytes at the site of infection. *L. major-*infected *Pdl1*^−/−^ mice expressed lower levels of MHCII and higher levels of CD206 on macrophages and monocytes and, more importantly, the lack of PD-L1 contributed to a reduced frequency of CD4^+^Ly6C^hi^ T effector cells and an increase of CD4^+^Foxp3^+^ regulatory T cells at the site of infection and in draining lymph nodes. Additionally, the lack of PD-L1 was associated with lower production of IL-27 by infiltrating monocytes and lower levels of the Th1 cytokines IFN-γ and TNF-α produced by CD4^+^ T effector cells. *Pdl1*^−/−^ mice initially exhibited larger lesions despite having a similar parasite load. Our results describe for the first time how the interruption of the PD-1/PD-L1 axis influences the immune response against CL and suggests that this axis regulates the balance between CD4^+^Ly6C^hi^ T effector cells and CD4^+^Foxp3^+^ regulatory T cells.

## Introduction

Cutaneous leishmaniasis (CL) is an important neglected tropical disease of the skin affecting between 600,000 and 1 million people per year (1, 2). Disease presentation ranges from the development of ulcers, which can spontaneously heal, to single or multiple chronic non-healing lesions, which are difficult to resolve (3). So far, drugs to control the disease are limited due to high costs and toxicity, and there is currently no safe and effective vaccine for application in humans (4, 5). The complexity of the host immune response to *Leishmania* is also a key limiting factor for developments in this field (6–8). Briefly, the response is initiated with the injection of *Leishmania* in the skin through a bite of an infected sand fly and subsequent infiltration of inflammatory cells (9, 10). Ultimately, parasites are phagocytized and end up replicating in dermal macrophages, while in local draining lymph nodes (dLN) Type I CD4^+^ T cells producing interferon (IFN)-γ are induced and enhance the respiratory burst of macrophages to eliminate parasites (11). However, even after lesion healing some parasites can persist. This sustains a population of short-lived CD4^+^Ly6C^+^ T effector cells that can rapidly migrate to the infected site upon re-challenge and produce high levels of IFN-γ (12). In mouse models of infection, expression of Ly6C has been used to identify highly differentiated CD4^+^ T effector cells (13–15). Ly6C expression on CD4^+^ T cells is mainly induced by IL-27 (16–18). In contrast, CD8^+^ T cells have been associated with stronger inflammation, increased TNF-α and IL-1β production, tissue damage, and disease severity, but none of these mechanisms control *Leishmania* replication (19–22). Furthermore, there is evidence that CD4^+^Foxp3^+^ regulatory T cells (Treg) play a crucial role in limiting the immune response, preventing tissue damage and promoting memory through interleukin (IL)-10 secretion (23–25).

There are still many gaps in the knowledge of how a protective CD4^+^ T cell response against *Leishmania* is activated, maintained, and controlled (26). In the last years it has been demonstrated that the expression of co-inhibitory receptors on activated T cells during the course of an immune response plays a pivotal role in dampening T cell activity (27–29). Moreover, blocking antibodies against some of those receptors, such as programmed cell death (PD)-1 (CD279) and programmed cell death ligand (PD-L)-1 (CD274, B7-H1), expressed by T cells and antigen presenting cells (APCs), respectively, could restore T cell function in skin cancer (30, 31). However, the role of the PD-1/PD-L1 axis during acquisition of immunity against many infectious agents is still not well-established, and this pathway may even suppress immunity, allowing chronic infections (16, 31, 32). To date, it has been shown in different murine infection models of *Leishmania* that blocking the PD-1/PD-L1 axis restores T cell function, resulting in increased IFN-γ production and diminished parasite burden (33–36). However, it is not clear how the PD-1/PD-L1 axis modulates CD4^+^ T cells during infection with *Leishmania*. In addition, human data is scarce and little is known about the expression of PD-1 by different CD4^+^ T cell subsets during chronic parasitic infections such as CL. Therefore, a better understanding of this pathway is of utmost importance to enhance the development of new therapeutic concepts.

To provide better insight on the role of the PD-1/PD-L1 axis during CL, we initially investigated PD-1 expression on CD4^+^ T cells from different patient cohorts and healthy individuals. Additionally, using a murine model of CL, we determined the cell types that expressed PD-1 and PD-L1 upon infection and analyzed the immune response of PD-L1-deficient mice (*Pdl1*^−/−^) after infection with *L. major*. Our data describe for the first time the immunological consequences of interrupting the PD-1/PD-L1 axis during the immune response against CL and provide evidence that this axis as an important regulator of CD4^+^Ly6C^hi^ T effector cells and CD4^+^Foxp3^+^ regulatory T cells during CL.

## Materials and Methods

### Ethics

C57BL/6J and *Pdl1*^−/−^ mice were bred in the animal facility of the Bernhard Nocht Institute for Tropical Medicine. Co-housed mice were used for comparison of wild-type (WT) and *Pdl1*^−/−^. All animal experiments were performed according to the German animal protection law under the supervision of a veterinarian. The responsible federal health authorities of the state of Hamburg (Behörde für Gesundheit und Verbraucherschutz) reviewed and approved the experimental protocol under the permission number N 027/2018. All mice were euthanized by cardiocentesis under deep CO_2_ narcosis, followed by confirmation through cervical dislocation. All animals were maintained in ventilated cages under low pathogen conditions. Age-matched (8–12 weeks old) female mice were used. IAM/FIOCRUZ Research Ethics Committee (Recife, Brazil) have approved the experimental protocols for this research (CAEE number 11083812.7.0000.5190). Selected individuals were invited to participate in the research and signed the “Term of Free and Informed Consent.” The selection of these individuals was based on criteria such as: being more than 12 years old, having confirmed diagnosis by the IAM/FIOCRUZ Leishmaniasis Reference Centre and being active lesion carriers. Our study group consisted of 10 patients with active CL and 10 patients post-treatment from Pernambuco rural endemic areas with one to four lesions and a disease with a mean duration of 50 days (15 days−3 months). Patients were submitted to blood collection after chemotherapy treatment with Glucantime® and clinical cure. The control group consisted of 10 healthy individuals from non-endemic areas with no previous history of CL and no prior blood transfusion. Patients and controls had a similar age range. Tables 1, 2 summarize clinical and laboratorial characteristics of active disease and of post-treatment patients, respectively.

**Table 1:** Characteristics of active disease patients.

**Patient**	**Sex**	**Age**	**Lesion localization**	**Number of lesions**	**Lesion dimensions (cm)**	**Leishmania search**	**PCR**
1	F	55	Left leg	3	3.0 × 3.0/1.5 × 1.5/1.5 × 1.0	+	+
2	M	30	Left and right arms	2	2.0 × 2.0/1.0 × 0.5	+	+
3	F	20	Left forearm	1	1.5 × 1.5	ND	+
4	F	18	Left leg	1	1.5 × 1.5	+	+
5	F	29	Left big toe	1	0.5 × 0.5	ND	+
6	M	58	Neck	1	3.0 × 3.0	+	+
7	M	42	Face	1	1.5 × 1.2	+	+
8	F	18	Right fist	1	5 (mm)	+	+
9	F	50	Right and left ankles	2	2.0 × 1.5/2.0 × 1.0	+	+
10	M	37	Left arm	1	2.0 × 2.0	+	ND

*ND, Non-determined*.

**Table 2:** Characteristics of post-treatment patients.

**Patient**	**Sex**	**Age**	**Scar localization**	**Number of scars**	**Scar dimensions (cm)**	**Months post-treatment at the time of blood collection**
1	M	34	Left leg	1	4.0 × 3.0	5
2	F	20	Left leg	1	4.5 × 2.0	2
3	F	30	Fist	1	8.0 × 2.0	4
4	M	18	Leg	1	2.0 × 2.0	4
5	M	18	Left leg	1	4.5 × 3.0	5
6	F	34	Ankle	1	7.0 × 2.5	2
7	F	36	Right leg	1	8.5 × 6.0	5
8	M	18	Right leg	2	6.5 × 3.0	5
9	M	18	Left arm	1	3.5 × 4.0	5
10	M	33	Right leg	1	4.0 × 4.0	4

### Human Blood Collection and Peripheral Blood Mononuclear Cell Isolation

Forty milliliters of peripheral blood were collected in heparinized tubes, diluted in a proportion of 2:1 (v/v) in PBS (pH 7.2), and then added to a Ficoll-Hypaque (Amersham Biosciences, Uppsala, Sweden) solution. After centrifugation at 400 × g for 30 min at 20°C, a ring of peripheral blood mononuclear cells (PBMCs) was obtained. Cells were washed twice with phosphate buffer solution (PBS) (pH 7.2) and submitted to centrifugation (300 × g for 10 min at 20°C). PBMCs were resuspended with 2 ml of RPMI 1640 medium (Cultilab, Campinas, SP, Brazil) supplemented with 10% fetal calf serum (Cultilab, Campinas, SP, Brazil) and 1% antibiotics (100 UI/ml penicillin and 100 μg/ml streptomycin- Sigma, St. Louis, MO). Cell viability was evaluated with Trypan blue dye (Sigma, St. Louis, MO), cells were counted in a Neubauer chamber, and the cell concentration was adjusted to 10^6^ cells for subsequent staining.

### Parasite Growth and Mice Infections

*Leishmania (Leishmania) major* (MHOM/IL/81/FE/BNI) were grown in complete (10% fetal calf serum, 5% penicillin-streptomycin, 5% glutamine) Schneider's Drosophila medium (PAN Biotech, Germany) at 27°C in an incubator under sterile conditions. Infectivity of parasites was maintained through animal passage and parasites were used for infection after maximum five passages in culture. Viable parasites were counted, washed with PBS (pH 7.0), and the concentration was adjusted to 3 × 10^6^ parasites in 10 μL volume, as described previously in other publications (37–39). Mice were kept under isoflurane/oxygen, the left ear was exposed, and 10 μL parasite-dose were injected.

### Processing of Ear and Lymph Node Tissues

Samples were collected and processed to obtain single cell suspensions. The dorsal and ventral ear layers were separated with forceps and individually deposited on wells of a 24-well bottom plate supplemented with RPMI 1640 containing 250 μg/mL Liberase^TM^ TL Research Grade (Roche) for 90 min at 37°C and 5% CO_2_. The reaction was stopped with 1 mL/well of cold media and the tissues were transferred to the top of a 70 μm mesh and mashed with the aid of a syringe piston. Cells were washed twice with PBS (pH 7.4) 2% FCS and finally resuspended with 1 mL of supplemented RPMI 1640 media. Lymph nodes were processed similarly.

### Lesion Size and Limiting Dilution Parasitaemia Analysis

An aliquot of 200 μL from each infected sample was used to perform a limiting dilution assay to quantify the parasite levels on ears and lymph nodes. Samples were centrifuged at 400 × g for 5 min and resuspended in 200 μL of complete Schneider's Drosophila medium. Samples were individually deposited on 96-well flat bottom plates and a serial dilution of 1:10 (v/v) was performed. Plates were incubated at 27°C for minimum of 7 days. After this period, the number of viable parasites in the tissues was calculated accordingly: [(Geo Mean of the duplicate from highest dilution)/lesion weight] × 50 (1,000/20–20 μL was initially used from 1,000 μL suspension) (40, 41).

### Antibodies, Flow Cytometry, and Chemokine Detection

Antibodies (α) were purchased from Biolegend (Germany) and BD Biosciences, unless noted otherwise. Mouse cells—The following antibodies were used for flow cytometry: αCD3-BUV395 (Clone 1 45-2C11) αCD4-V500 (Clone RM4-5), αCD44-AF700/BV421(Clone IM7), αCD62L-APC/Cy7 (Clone MEL-14), αCD27-FITC/PerCP Cy5.5 (Clone LG3A.10), αLy6C-PE/BV500/PerCP-Cy5.5 (Clone HK1.4), αFoxp3-APC (Clone FJK 16s), αIFN-γ-AF488 (Clone XMG 1.2), αTNF-α-PECy7 (Clone MP6 XT22), αPD-1-PECy7 (Clone RMP1-30), αIL-27-PE (Clone MM27-7B1), αCD45-PECy7 (Clone 30-F11), αCD11b-APC Cy7 (Clone M1/70), αF4/80-AF700 (Clone BM8), αPD-L1-PE/PE/Cy7/APC (Clone 10F.9G2), αMHC Class II-BV510 (Clone M5/114.15.2), αCD86-PE/Cy7 (Clone GL1), αCD206-PE/Dazzle (Clone C068C2), αRelm Alpha (Polyclonal rabbit IgG—Genetex), αRabbit IgG-BV421 (Clone Poly4064), αYm1 (Polyclonal goat IgG—R&D Systems), αGoat IgG-FITC (Polyclonal—Invitrogen). Approximately 10^6^ cells were deposited individually per well in 96-well round bottom plates for antibody staining. Cells were washed twice with PBS (pH 7.2) 2% FCS, 300 × g for 10 min at RT. Cells were stained with cell surface antibodies diluted in Fc Block solution for 1h at 4°C. After this period, plates were washed twice with 200 μL per well of PBS (pH 7.2) 2% FCS, 300 × g for 10 min at RT, and cells were fixed with 200 μL per well of 1x fixation buffer (eBioscience Foxp3 staining buffer) for 30 min. Cells were washed twice with 1× permeabilization buffer (eBioscience Foxp3 staining buffer), 300 × g for 10 min at RT. Cells were stained with intracellular antibodies diluted in permeabilization buffer and incubated for 1h at 4°C. Afterwards cells were washed twice with 1× permeabilization buffer (eBioscience Foxp3 staining buffer), 300 × g for 10 min at RT. Stained cells were resuspended in an appropriate volume of flow cytometry staining buffer or paraformaldehyde 1% prior to FACS analysis. For intracellular cytokine staining, cells in RPMI 1640 containing GolgiStop (BD Biosciences) diluted 1:1,000 (v/v) were deposited in 96-well round bottom plates and incubated for 4–5 h at 37°C with 5% CO_2_. For macrophage staining with antibodies, cells were blocked with 50 μL of blocking solution (PBS pH 7.2, 2% FCS, 1:100,000 of CD16/CD32) for 15 min at 4°C. For staining with purified antibodies, an extra cell surface staining step for detection with fluorochrome-conjugated anti-IgG was added. Samples were acquired on a BD LSRII (BD Biosciences) and the generated data were analyzed with the current version of FlowJo Software (v10.6.2).

Antibodies (α) were purchased from Biolegend (Germany). Human cells—The following antibodies were used for flow cytometry: αCD3-APC Cy7 (Clone HIT3a), αCD4 BV605 (Clone A161A1), αCD45RO-PE/Cy7 (Clone UCHL1), αCD197/CCR7-BV421 (Clone G043H7), and αPD-1-APC (Clone EH12.2H7). Cells from patients were individually deposited in FACS tubes and stained with cell surface antibodies for 30 min at 4°C, protected from light. After this period, cells were washed twice with PBS (pH 7.2) and submitted to centrifugation (300 × g for 10 min at 20°C). Cells were fixed with 1× fix/perm buffer solution (eBioscience) prior to FACS acquisition. Samples were acquired on a BD FACS Aria III (BD Biosciences) and the generated data were analyzed with FlowJo Software (v10.6.2).

For chemokine detection, single cell suspensions from the ears of infected mice were deposited in 96-well round bottom plates and incubated for 24h at 37°C with 5% CO_2_. MCP-1 (CCL2) chemokine detection was performed by ELISA following manufacturers' recommendations (Biolegend).

### Statistical Analysis

Statistical analysis was performed using Graph Pad Prism 8. The statistical significance between the two groups was calculated using the two-tailed Mann-Whitney test. Statistical assumptions were made at 95% confidence level and ^*^p < 0.05, ^**^p < 0.01, ^***^p < 0.001 and ^****^p < 0.0001.

## Results

### PD-1 Is Upregulated on Different Human CD4^+^ T Cell Subsets During Cutaneous Leishmaniasis

**Figure 1:**
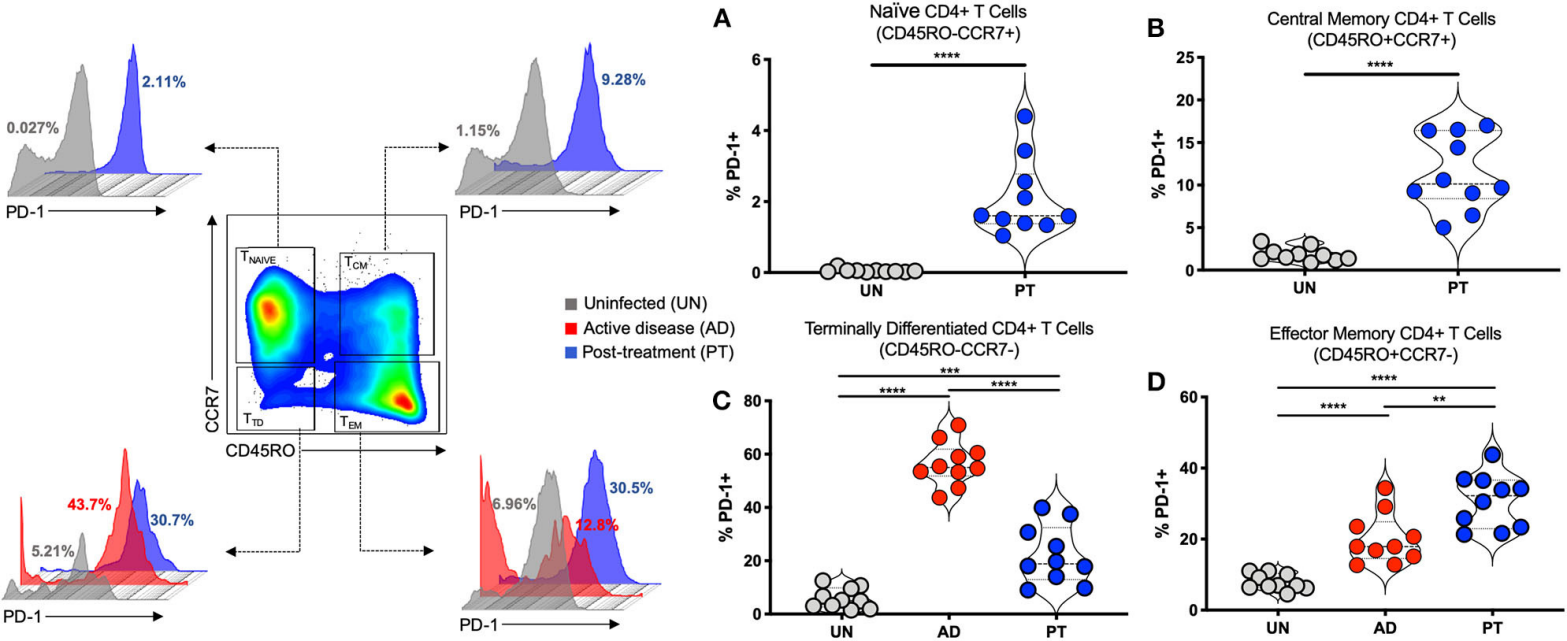
PD-1 is upregulated on different human CD4^+^ T cell subsets during cutaneous leishmaniasis. Representative flow cytometry graphs and independent data depicting the frequency (%) of PD-1^+^ in Naïve CD4^+^ T cells (CD45RO^−^CCR7^+^) **(A)**, Central Memory CD4^+^ T cells (CD45RO^+^CCR7^+^) **(B)**, Terminally Differentiated CD4^+^ T cells (CD45RO^−^CCR7^−^) **(C)**, and Effector Memory CD4^+^ T cells (CD45RO^+^CCR7^−^) **(D)** in the peripheral blood of patients with active lesions before treatment (Active disease—AD, *n* = 10) and patients less than 6 months after clinical cure of CL (Post-treatment—PT, *n* = 10), when compared to control group of healthy individuals (Uninfected—UN, *n* = 10). Mean ± SD (Error bars). **p < 0.01, ***p < 0.001 and ****p < 0.0001, Mann-Whitney *U* rank sum test.

Based on the expression of CD45RO and CCR7, human CD4^+^ T cells can be divided into four different subsets: CD45RO^−^CCR7^+^ T naïve (T_NAÏ*VE*_), CD45RO^+^CCR7^+^ T central memory (T_CM_), CD45RO^+^CCR7^−^ T effector memory (T_EM_), and CD45RO^−^CCR7^−^ T terminally differentiated (T_TD_) T cells (42–44). The frequency of PD-1^+^ T cells was analyzed in those CD4^+^ T cell subsets. We observed high frequencies of CD4^+^PD-1^+^ T cells in T_CM_ of post-treated (PT) patients compared to uninfected individuals (Figures 1A,B). Active CL disease (AD) patients displayed very low frequencies of T_NAÏ*VE*_ and T_CM_ cells. However, higher frequencies of CD4^+^PD-1^+^ T cells were observed in T_TD_ cells of active CL compared to uninfected individuals and PT patients (Figure 1C). Finally, increasing frequencies of CD4^+^PD-1^+^ T cells were observed in T_EM_ cells of AD and PT patients (Figure 1D). One interesting aspect of this result is the continuously increasing frequency of PD-1^+^ T cells with increasing development of CD4^+^ T cell subpopulations, with a very low frequency among T_NAÏ*VE*_ and very high frequency among T_TD_ cells. Moreover, during AD, higher frequencies of PD-1^+^ T cells were observed in T_TD_ cells compared to the other subsets. After treatment (PT), the frequency of PD-1^+^ T cells declined in T_TD_ cells but was still high in T_EM_ cells. To further study PD-1 and PD-L1 expression at different sites and on different cell types we decided to employ a murine model of CL.

### PD-1 and Its Ligand PD-L1 Are Upregulated by Different Cell Types in the Lesion of *Leishmania major*-Infected Mice

**Figure 2:**
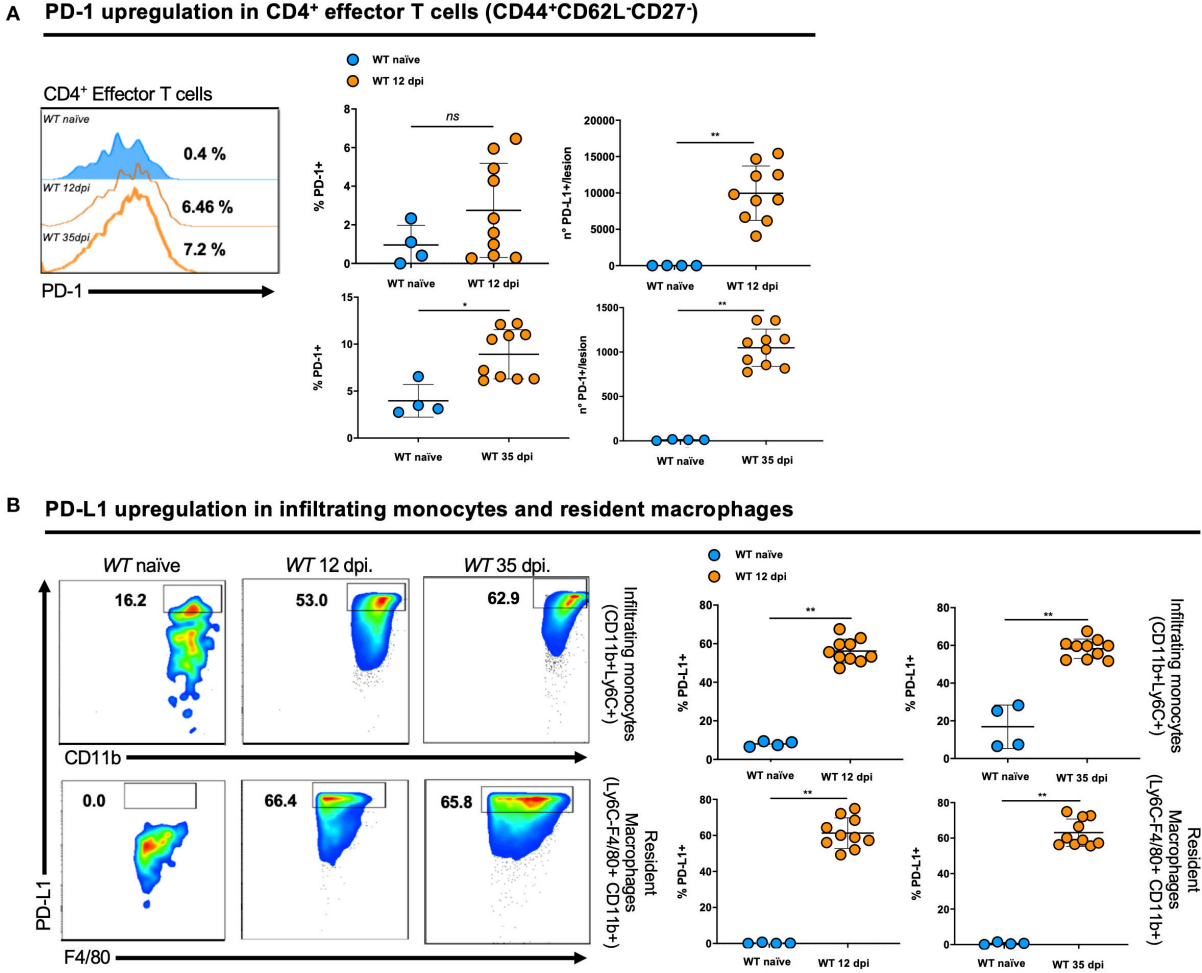
PD-1 and its ligand PD-L1 are upregulated by different cell types in the lesion of *Leishmania major*-infected mice. **(A)** Representative flow cytometric analysis and summary of data of PD-1 levels in activated CD4^+^ T effector cells (CD3^+^CD4^+^CD44^+^CD62L^−^CD27^−^) isolated from the ear of naïve and 12 and 35 days post-infections (dpi) Wild-type (WT) mice. **(B)** Representative flow cytometry graphs and summary of data of PD-L1 levels in infiltrating monocytes (CD45^+^Ly6C^+^CD11b^+^) and resident macrophages (CD45^+^Ly6C^−^CD11b^+^F4/80^+^) isolated from the ear of naïve and 12 and 35 dpi WT mice. Results are representative of two independent experiments with 2–5 mice/group. Mean ± SD (Standard deviation bars). *p < 0.05 and **p < 0.01, Mann-Whitney *U* rank sum test.

The expression of PD-1 on activated CD4^+^ T effector cells (CD3^+^CD4^+^CD44^+^CD62L^−^CD27^−^) (Figure 2A) and of PD-L1 on infiltrating monocytes (CD45^+^Ly6C^+^CD11b^+^) and resident macrophages (CD45^+^Ly6C^−^CD11b^+^F4/80^+^) (Figure 2B) were assessed in tissues from naïve WT mice and WT mice on days 12 and 35 post-infection (dpi). We observed a strong induction of PD-1 on activated CD4^+^ T effector cells at both time points compared to naïve WT mice. In parallel, we detected PD-L1 on infiltrating monocytes and resident macrophages isolated from the infected ear of *L. major-*infected mice compared to uninfected controls. This finding suggests that the PD-1/PD-L1 axis may have important regulatory functions during infection by *Leishmania* and corroborates our findings on PD-1 induction on CD4^+^ T cells from infected patients. Thus the PD-1/PD-L1 axis, in particular the inhibition of T cell function, might be important to control the degree of CD4^+^ T cell activation. Thus, we decided to evaluate the immune response of *Pdl1* knockout (*Pdl1*^−/−^) mice during *Leishmania* infection.

### Infiltrating Monocytes and Resident Macrophages From *Pdl1*^-/-^ Mice Exhibit a Weaker Inflammatory Phenotype After *Leishmania major* Infection

**Figure 3:**
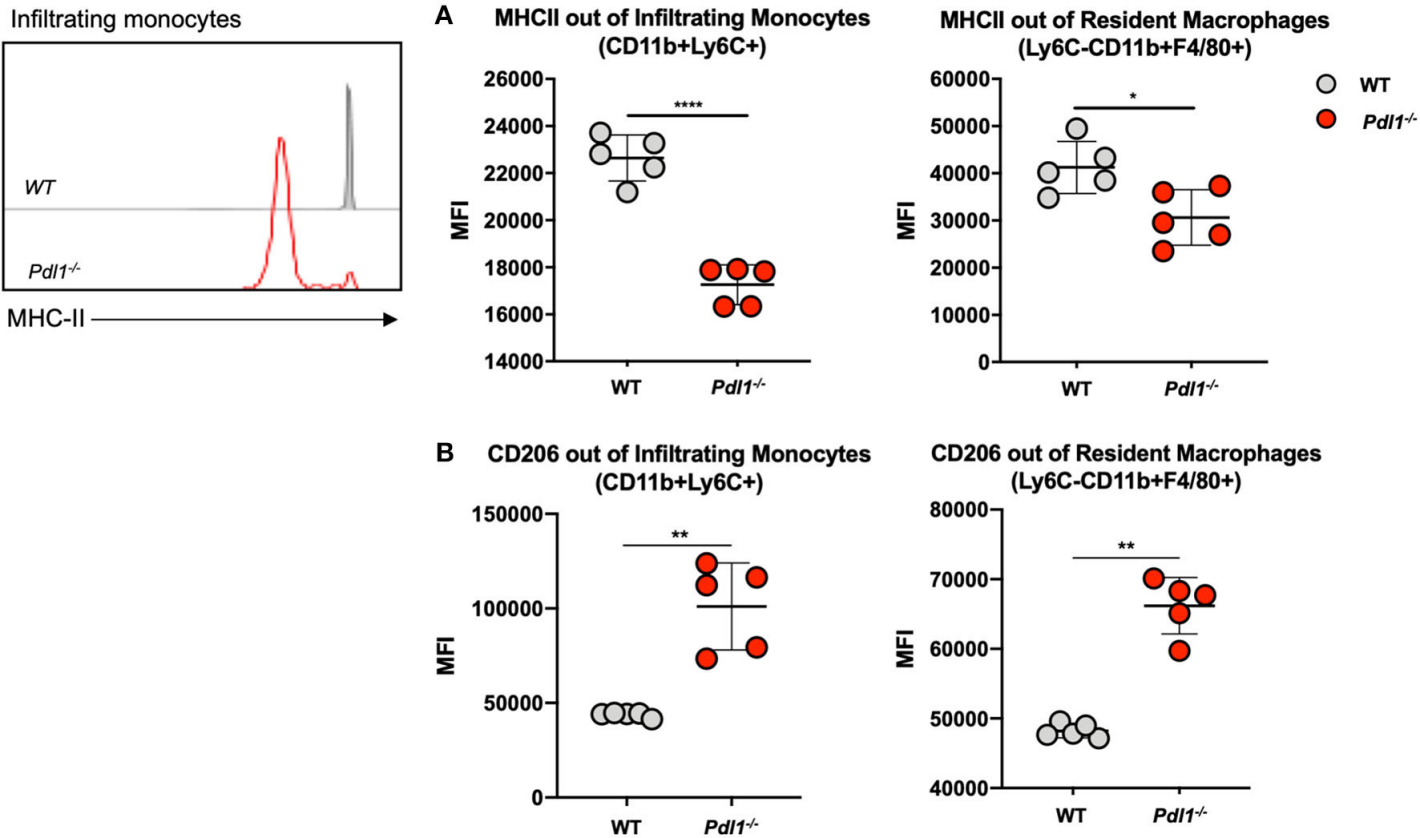
Infiltrating monocytes and resident macrophages from *Pdl1*^−/−^ mice exhibit a weaker pro-inflammatory phenotype after *Leishmania major* infection. Representative flow cytograms and summary of data of MHC Class II **(A)** and CD206 **(B)** expression on infiltrating monocytes and resident macrophages from the *Leishmania major* infected ear of Wild-type (WT) and *Pdl1*^−/−^ mice 35 dpi. Results are representative of one of two independent experiments with 5 mice/group. Mean ± SD (Standard deviation bars). *p < 0.05, **p < 0.01 and ****p < 0.0001, Mann-Whitney *U* rank sum test.

We investigated the activation status of infiltrating monocytes (CD45^+^Ly6C^+^CD11b^+^) and resident macrophages (CD45^+^Ly6C^−^CD11b^+^F4/80^+^) by analyzing the expression of major histocompatibility complex (MHC) class II and CD206 in the infected ear of *L. major*-infected *Pdl1*^−/−^ and WT mice at 35 dpi (Figure 3). We observed significantly lower levels of MHCII expression and higher levels of CD206 expression in samples derived from *L. major*-infected *Pdl1*^−/−^ mice compared to their WT counterparts. Interestingly, this result suggests that the lack of PD-1/PD-L1 signaling induces a weaker pro-inflammatory phenotype of PD-L1-deficient macrophages and monocytes. Based on this finding we further analyzed whether an impairment of the PD-1/PD-L1 axis might also affect the CD4^+^ T cell compartment.

### PD-L1 Is Required for the Expansion of CD4^+^Ly6C^hi^ Effector T Cells and CD4^+^Foxp3^+^ Regulatory T Cells in the Lesion of *Leishmania major*-Infected Mice and Draining Lymph Nodes

**Figure 4:**
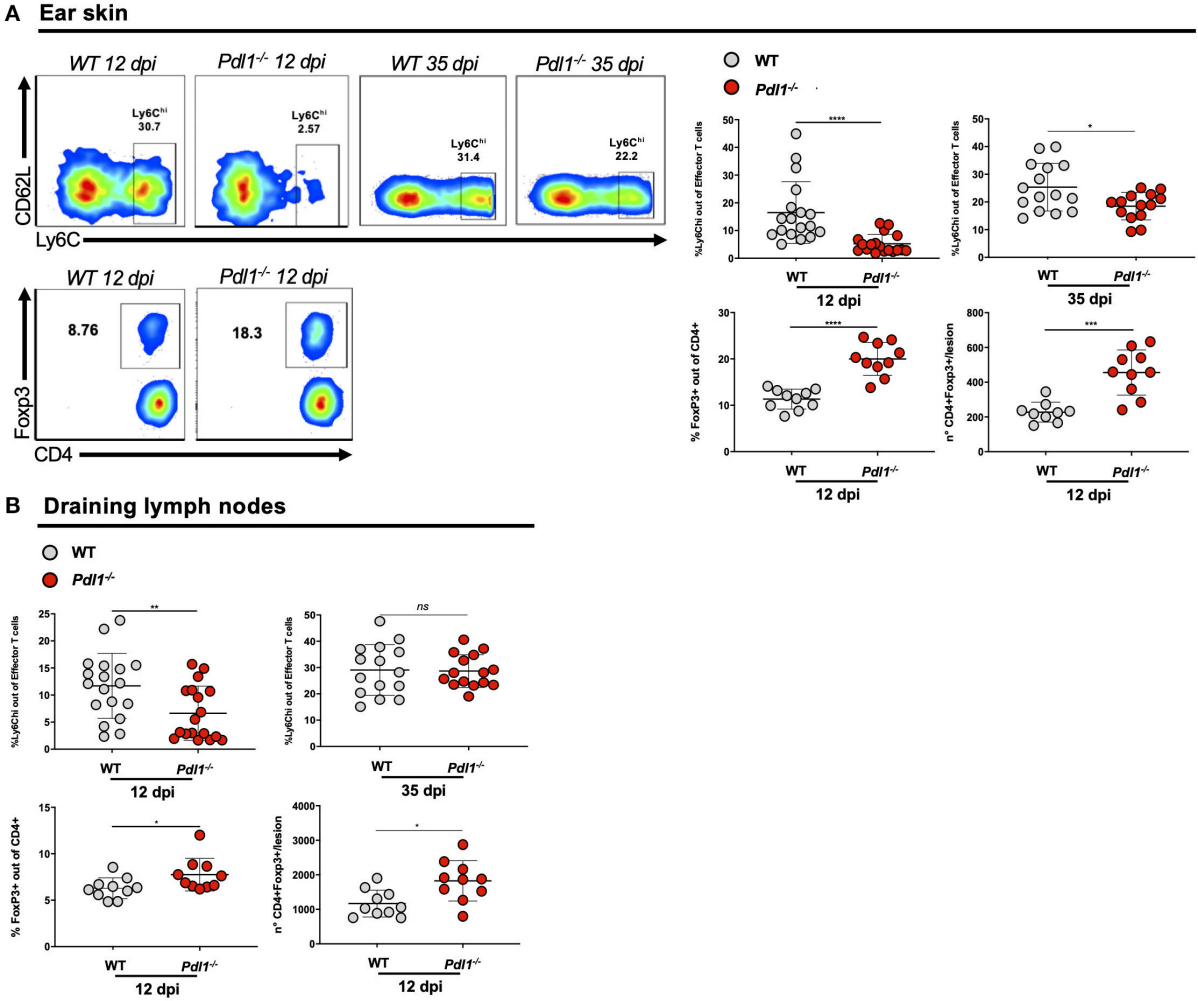
PD-L1 is required for the expansion of CD4^+^Ly6C^hi^ effector T cells and CD4^+^Foxp3^+^ regulatory T cells in the lesion of *Leishmania major*-infected mice and draining lymph nodes. **(A)** Representative flow cytometric dot-plots of the ear tissues analyses are shown and summary of data, highlighting the decreased expansion of CD4^+^Ly6C^hi^ effector T cells (12 and 35 dpi) and the increased expansion of CD4^+^Foxp3^+^ T cells (12 dpi) in *Pdl1*^−/−^ mice, compared to WT mice. **(B)** Summary of data of CD4^+^Ly6C^hi^ T effector cells (12 and 35 dpi) and CD4^+^ Foxp3^+^ T cells (12 dpi) in the lymph nodes of *Pdl1*^−/−^ mice, compared to WT mice. Results are representative of two or three independent experiments with 2–5 mice/group. Mean ± SD (Standard deviation bars). *ns* = no statistically significant, *p < 0.05, **p < 0.01, ***p < 0.001, ****p < 0.0001, Mann-Whitney *U* rank sum test.

Interestingly, we observed that *L. major* infection of *Pdl1*^−/−^ mice was accompanied by a reduced expansion of activated CD4^+^Ly6C^hi^ T effector cells and an increase in the percentage of CD4^+^Foxp3^+^ T regulatory cells (Treg) in the infected ear (Figure 4A) and in the ear draining lymph nodes (Figure 4B) of *Pdl1*^−/−^ compared to WT mice. Particularly striking is the difference of subpopulations of CD4^+^ T cells infiltrating the ear lesion, which shows that the lack of PD-L1 in a murine model of *L. major* infection results in a diminished number of CD4^+^Ly6C^hi^ effector T cells, but an increased number of Tregs.

### Lack of PD-L1 Is Associated With Lower Production of IL-27, IFN-γ, TNF-α and Increased Secretion of MCP-1/CCL2

**Figure 5:**
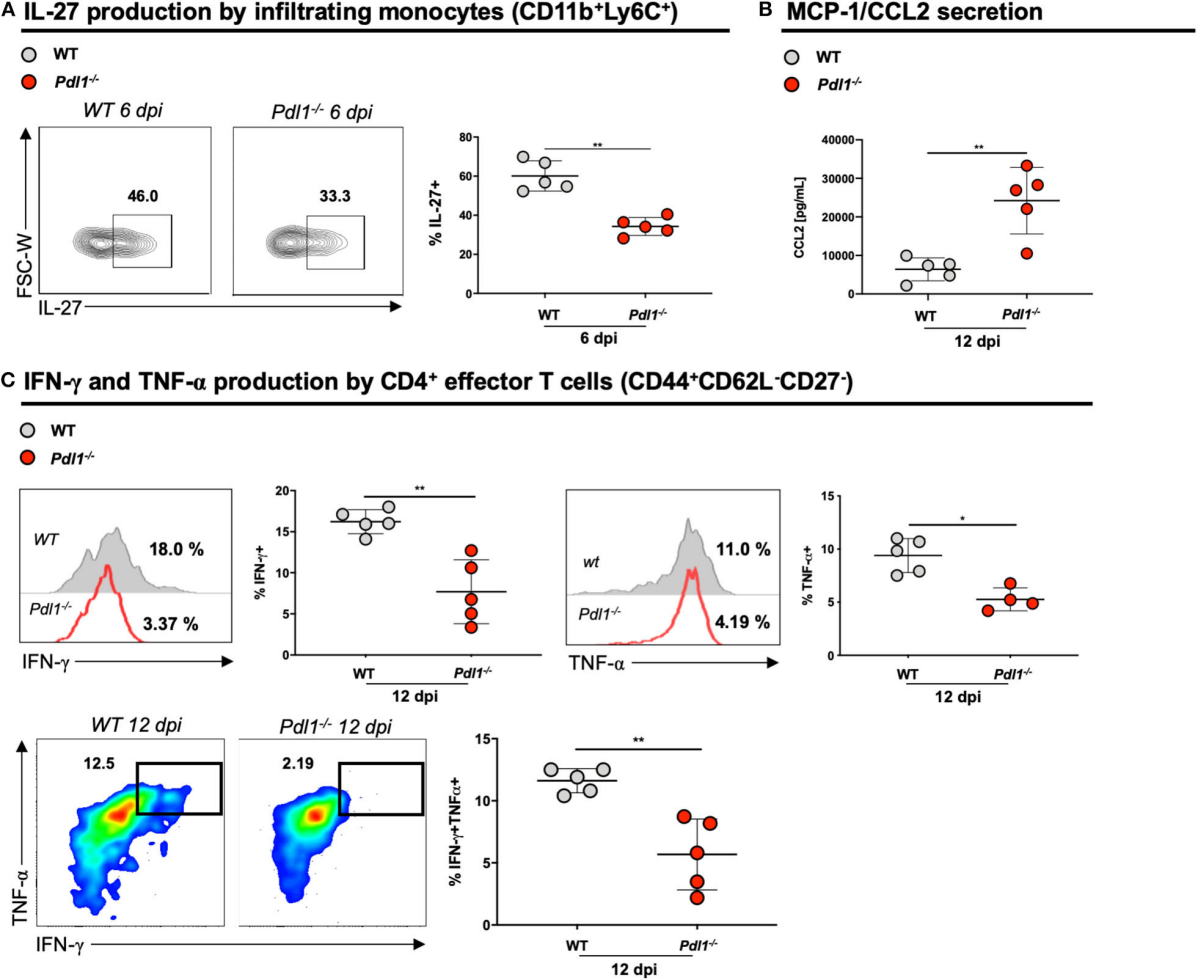
Lack of PD-L1 is associated with lower production of IL-27, IFN-γ, and TNF-α and increased secretion of MCP-1/CCL2. **(A)** Representative flow cytometric dot-plots and analyses of IL-27 production of infiltrating monocytes (CD11b^+^Ly6C^+^) from ear skin of Wild-type (WT) and PD-L1 deficient mice (*Pdl1*^−/−^) 6 dpi. **(B)** Analyses of CCL2 secreted in the ear skin cells of WT and *Pdl1*^−/−^ 12 dpi. **(C)** Representative histograms, dot-plots and analyses of IFN-γ, and TNF-α produced by effector CD4^+^ T cells from ear skin of WT and *Pdl1*^−/−^ 12 dpi. Results are representative of two or three independent experiments with 2–3. Mean ± SD (Standard deviation bars). *p < 0.05 and **p < 0.01, Mann-Whitney U rank sum test.

**Figure 6:**
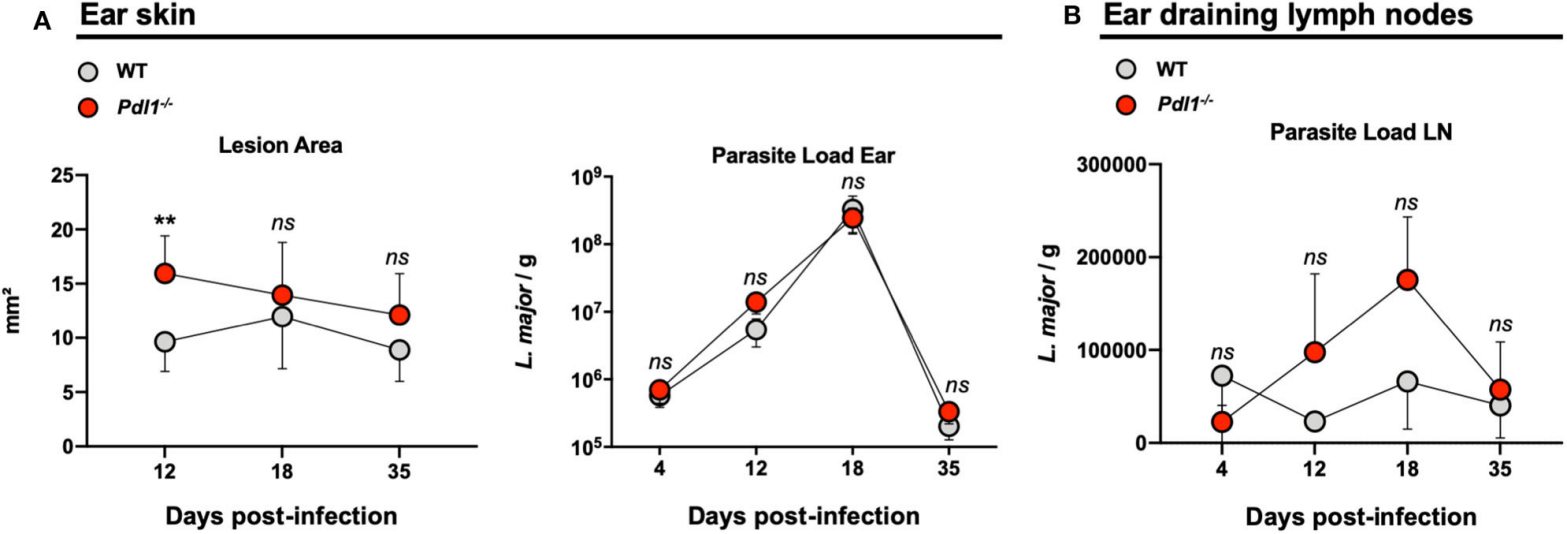
Lack of PD-L1 induced larger lesions after infection with *Leishmania major*, but did not affect parasite burden. **(A)** Summary of data representing the lesion area and parasite load of *Leishmania major* infected ear from Wild-type (WT) and *Pdl1*^−/−^ mice. **(B)** Independent data representing the parasite load of ear draining lymph nodes from WT and *Pdl1*^−/−^ mice. Results are representative of 2–3 independent experiments with 3–5 mice/group. Mean ± SD (Standard deviation bars). *ns* = no statistically significant, **p < 0.001, Mann-Whitney *U* rank sum test.

Surprisingly, we observed that the lack of PD-L1 is associated with lower production of IL-27 by monocytes infiltrating the infected skin 6 dpi (Figure 5A), but not by resident macrophages or other APCs. Among chemokines implicated in T cell recruitment, we observed an increased production of CCL2 in the infected ear of *Pdl1*^−/−^ mice compared to WT counterparts (Figure 5B). Moreover, CD4^+^ T effector cells from *Pdl1*^−/−^ mice produced lower levels of INF-γ and TNF-α 12 dpi (Figure 5C). This result suggests that the lower frequency of CD4^+^Ly6C^hi^ effector T cells might be due to the lower production of IL-27. Additionally, this result provides evidence that pro-inflammatory cytokines are not involved in the initial immunopathology driving increased lesion size (Figure 6).

### Lack of PD-L1 Induced Larger Lesions After Infection With *Leishmania major*, but Did Not Affect Parasite Burden

Parasite burden and lesion areas were assessed in *Pdl1*^−/−^ mice and their WT counterparts after *L. major* infection in the ear at different time points. However, with regard to the reduced frequency of activated CD4^+^Ly6C^hi^ T effector cells, the lesion area was larger in the *Pdl1*^−/−^ mice compared to the WT mice early after infection (12 dpi) (Figure 6A), although a similar number of parasites was observed in the two mouse strains at each time point analyzed (12, 18, 35 dpi) for both the ear (Figure 6A) and the dLN (Figure 6B). Interestingly, the difference in lesion area between WT and *Pdl1*^−/−^ was not observed at later time points, suggesting a role for PD-L1 on lesion development only in the initial phase of the response.

## Discussion

The regulation of T cell responses in leishmaniasis has been extensively investigated. The co-inhibitory receptor PD-1 is a potential target since it maintains immune homeostasis by inducing peripheral tolerance and protecting tissues from autoimmune attack (45). Moreover, blocking PD-1 or its ligand PD-L1 restores T cell function in different types of cancer, which is associated with a better disease outcome (46). However, despite its importance, the role of this axis during acquisition of immunity against CL is not well-established yet (27, 47, 48).

In the present study we found increasing frequencies of PD-1^+^ T cells in different subsets of CD4^+^ T cells from patients with acute and post-treatment CL. Very low numbers of PD-1^+^ T cells were observed in CD4^+^ T_NAÏ*VE*_ compared to the other subpopulations of CD4^+^ T cells like T_CM_, T_EM_, and T_TD_ cells. High numbers of PD-1^+^ T cells were observed in T cell compartments with more activated CD4^+^ T cells, such as T_CM_, T_EM_, and T_TD_. Moreover, patients with active disease display high numbers of PD-1^+^ T cells in CD4^+^ T_TD_, and in the post-treatment group the numbers of PD-1^+^ T cells are higher in CD4^+^ T_EM_ cells. It has been shown that antigen-specific T cells express PD-1 upon activation. This expression is tightly regulated and it remains highly expressed during chronic antigen exposure (49, 50). There are very few data about the differential expression of PD-1 in subpopulations of naïve, effector, and memory CD4^+^ T cells, and most of the data associates PD-1 expression with T cell exhaustion (51–53). Activated CD4^+^ T cells play an important role during human CL, acting as an important source of cytokines such as IFN-γ and TNF-α that are associated with lesion healing or disease progression (54, 55). This result suggests that PD-1 is induced on CD4^+^ T cells and potentially counter-regulates the immune response. However, the exact role of PD-1 expression on CD4^+^ T cells in the different subpopulations needs to be studied intensively. In addition, the high numbers of CD4^+^PD-1^+^ T cells which remained in patients after treatment might be an indirect indication of either a prolonged antigen exposure or a state of exhaustion that persists even after antigen removal. Lesion healing in clinically cured CL patients does not necessarily correlate with parasite clearance since the patients can suffer from reoccurrence of lesions in distinct and multiple numbers (56). Thus, our data suggest that PD-1/PD-L1 interactions are likely to occur during the course of human CL and persist post-treatment, which might contribute to the modulation CD4^+^ T cell activity.

To further study the function of PD-1 we employed an infection model using resistant C57BL/6 mice to determine which cell types express PD-1 and its ligand PD-L1 upon *L. major* infection at different time points. Furthermore, using full body *Pdl1*^−/−^ mice we observed that the lack of PD-L1 impacts the response of CD4^+^ T cells by reducing the frequency of CD4^+^ T effector cells and by increasing the frequency of CD4^+^Foxp3^+^ regulatory T cells, which affects the production of cytokines and chemokines. Nevertheless, *Pdl1*^−/−^ mice displayed larger lesions in the initial phase of infection, but displayed no differences in the number of parasites, which highlights the importance of PD-1 in the initial phase of the immune response against *Leishmania*.

During murine infection by *L. major*, increased frequencies of activated CD4^+^PD-1^+^ T effector cells were found at the infected site and in draining lymph nodes. This PD-1 expression is possibly a result of T cell activation, since *Leishmania* infection results in IL-12-dependent differentiation and activation of *Leishmania-*specific CD4^+^ T cells producing mainly type I cytokines (IFN-γ, TNF-α, IL-2) to activate macrophages and eliminate parasites (55). Moreover, it was previously demonstrated in *Leishmania* infection of human and mice that an impairment of CD8^+^ T cell function is associated with PD-1 expression, and the immunization of mice with lipophosphoglycan from *Leishmania* induces a dose-dependent expression of PD-1 on CD8^+^ T cells, but not on CD4^+^ T cells (29, 57–59). Our data highlights that PD-1 is induced on CD4^+^ T cells during *Leishmania* infection, which may counterbalance excessive activation when binding to PD-L1. We found high levels of PD-L1 expression on infiltrating monocytes and tissue-resident macrophages during *L. major* infection. These two cell types are particularly involved in the immune response against *Leishmania*, and T cell-derived IFN-γ is one of the most important cytokines that induces PD-L1 expression (60). However, it is not clear whether those levels of PD-L1 are solely induced by cytokines or if intrinsic *Leishmania* factors contribute to PD-L1 expression as a parasite-induced immune escape mechanism (61). Recent studies indicate that blocking PD-L1 in VL models restores CD4^+^ and CD8^+^ T cell function and leads to an increased survival of CD8^+^ T cells (29, 35, 62, 63). However, immune regulation in the skin might be different compared to other organs.

Upon *L. major* infection, resident macrophages and infiltrating monocytes from *Pdl1*^−/−^ mice are less activated compared to those from WT mice. This is in contrast with the current idea that PD-1/PD-L1 interaction dampens the immune response. Indeed, our data suggest that the lack of PD-L1 in resident macrophages and infiltrating monocytes dampen their pro-inflammatory phenotype, which would be necessary to eliminate intracellular *Leishmania*.

Lower frequencies of activated CD4^+^Ly6C^hi^ effector T cells and higher levels of CD4^+^Foxp3^+^ regulatory T cells were observed in *Pdl1*^−/−^ mice infected with *L. major*. Previously, it was shown that short-lived CD4^+^Ly6C^+^ effector T cells are capable of producing more IFN-γ compared to other cell subsets upon *Leishmania* infection (12). This study suggests that PD-1/PD-L1 engagement is required to control the expansion of this population of CD4^+^Ly6C^+^effector T cells. A possible scenario for this might be concluded from a recent study showing that PD-L1 and CD80 on APCs are interacting in *cis* and thus preventing the interactions of PD-1 and PD-L1 *in trans* (64). Then, a lack of PD-L1 on APCs would allow the interaction of CD80 with CTLA-4, which might lead to a reduced frequency of CD4^+^ T effector cells.

The PD-1/PD-L1 pathway also regulates the development and function of Treg by promoting Foxp3 expression (31). However, it was shown recently that a blockade of PD-1 can increase the proliferation and suppressive capacity of Tregs in inflamed tissues (65, 66). Therefore, it is tempting to speculate that the lack of PD-L1 leads to an increased expansion of CD4^+^Foxp3^+^ Treg cells and thereby contributes to a diminished expansion of CD4^+^Ly6C^hi^ T effector cells.

IL-27 was shown to be a major cytokine that induces Ly6C expression on CD4^+^ T cells upon TCR engagement (16, 67, 68). Our data indicate that the lack of PD-L1 is associated with a lower production of IL-27 by monocytes infiltrating the lesion during the initial phase of infection, which might lead to a lower amount of CD4^+^Ly6C^hi^ T effector cells and a diminished secretion of Th1 cytokines.

We found no differences in the parasite load in the tissues of *Leishmania-*infected WT mice and *Pdl1*^−/−^ mice. However, we observed larger lesions in the latter. Parasite clearance has been associated with activation of macrophages by CD4^+^ T effector cells producing IFN-γ. Larger lesions might be the result of an immunopathology rather than by the parasite itself. However, the lower levels of IFN-γ and TNF-α produced by CD4^+^ T effector cells in *Pdl1*^−/−^ mice suggest that these pro-inflammatory cytokines are not driving lesion formation. Other immune pathways, like CD8^+^ T cells, might promote this inflammation by secreting TNF-α, IL-1β, or granzymes, which have been associated with disease severity of CL without altering parasite killing (19–22).

The present work demonstrates that the PD-1/PD-L1 axis has important functions during human CL and murine experimental infection. The lack of PD-L1 impairs the expansion of activated CD4^+^Ly6C^hi^ T effector cells during infection and enhances the frequency of Tregs in the infected ear and in the draining lymph nodes. This results in larger lesions but has no effect on parasite burden. Importantly, our data suggest that the PD-1/PD-L1 axis during CL provides a delicate fine-tuning of the immune response, which ensures an optimal anti-parasitic immune response without overwhelming inflammation.

## Data Availability Statement

All datasets generated for this study are included in the article/Supplementary Material.

## Ethics Statement

The studies involving human participants were reviewed and approved by CPqAM/FIOCRUZ Research Ethics Committee. The patients/participants provided their written informed consent to participate in this study. The animal study was reviewed and approved by Federal health authorities of the Hamburg (Behörde für Gesundheit und Verbraucherschutz).

## Author Contributions

RF and RG performed the experiments. RF and TJ were responsible for acquisition of data, analysis, interpretation, and drafting the manuscript. VP and MB were responsible for human leishmaniasis diagnosis, analysis, and for providing the laboratory and materials for human experiments. SC and HL helped with murine model for leishmania infection. LB was responsible for interpretation of data and provided reagents. All authors have read and approved the final manuscript. All authors contributed to the article and approved the submitted version.

## Conflict of Interest

The authors declare that the research was conducted in the absence of any commercial or financial relationships that could be construed as a potential conflict of interest.
