# Supplementary material for: Programmed Cell Death Ligand (PD-L)-1 Contributes to the Regulation of CD4+ T Effector and Regulatory T Cells in Cutaneous Leishmaniasis
Source: Front Immunol. 2020 Oct 22;11:574491. doi: 10.3389/fimmu.2020.574491 (PMC7642203; doi:10.3389/fimmu.2020.574491)

**Supplemental Material**

**Programmed cell death ligand (PD-L)-1 contributes to the regulation of CD4^+^ T effector and regulatory T cells in cutaneous leishmaniasis**

**Rafael de Freitas e Silva, Rosa Isela Gálvez, Valeria Rego Alves Pereira, Maria Edileuza Felinto de Brito,** **Siew Ling Choy, Hannelore Lotter, Lidia Bosurgi and Thomas Jacobs**

**Supplementary Figure 1. Flow cytometry data of human CD4+ T cells was analyzed according to the strategy shown.**

**
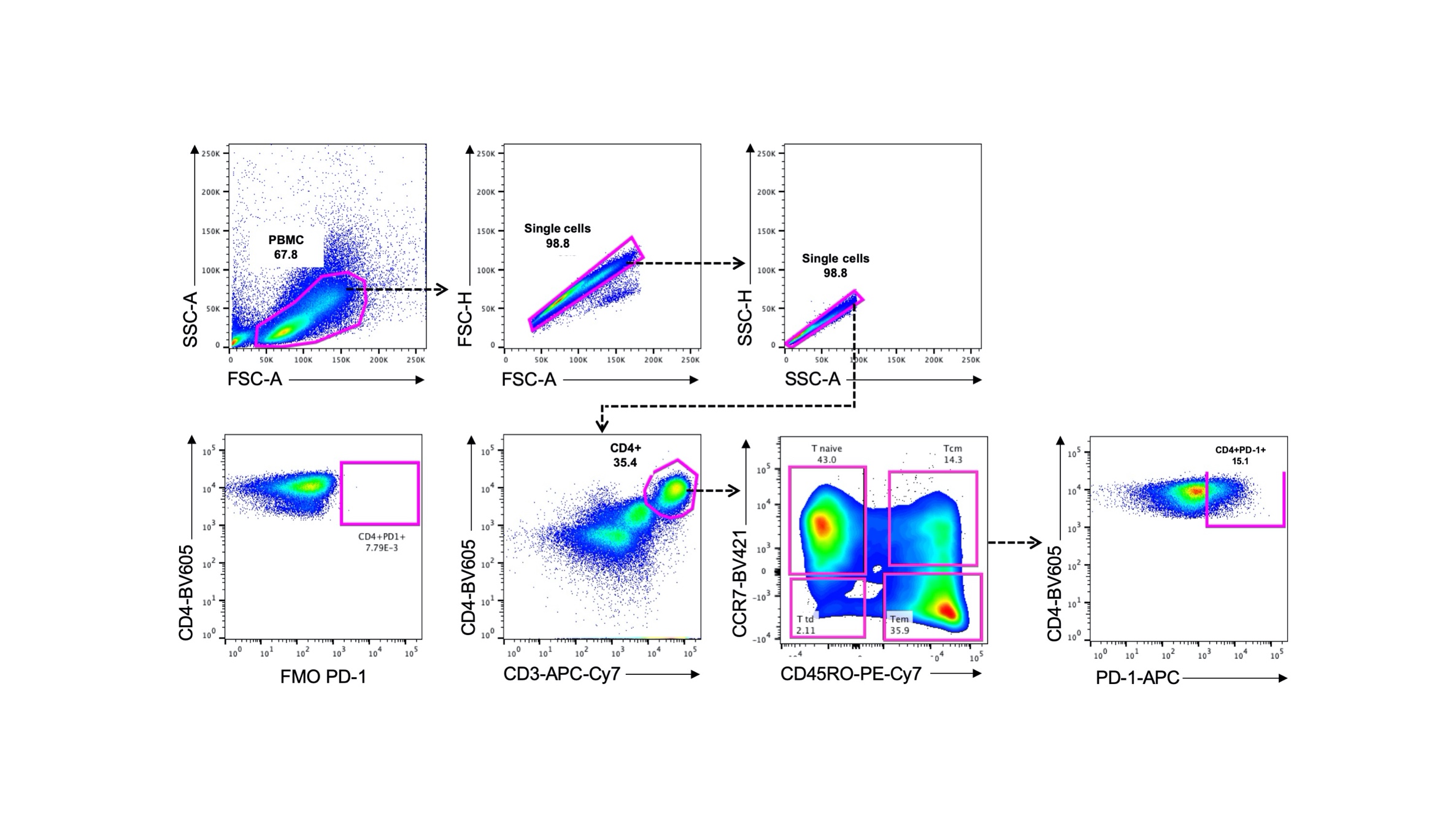
**

**Supplementary Figure 2. Flow cytometry data of murine effector CD4+ T cells was analyzed according to the gate strategy shown.**

**
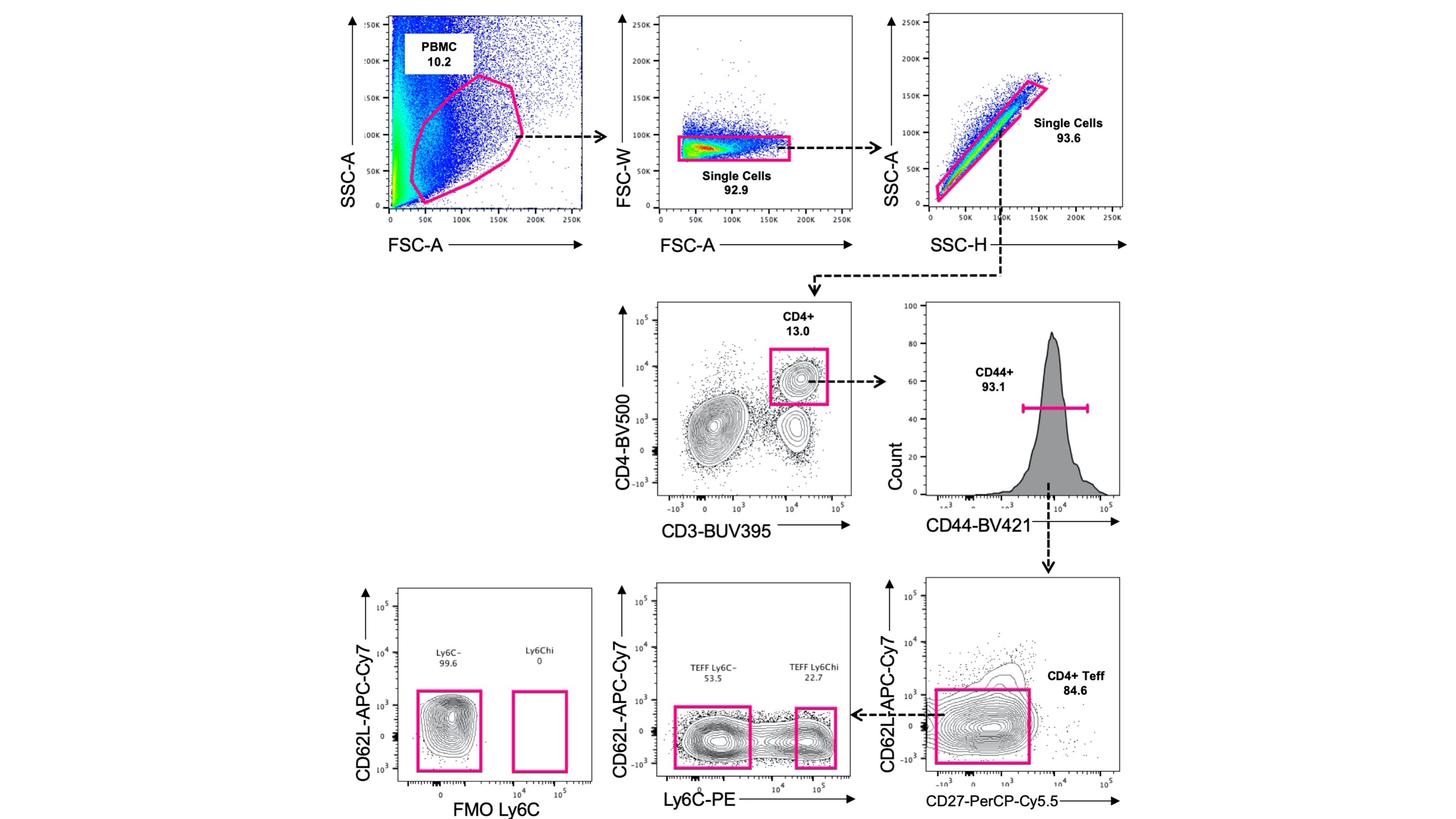
**

**Supplementary Figure 3. Flow cytometry data of murine macrophages and monocytes was analyzed according to the gate strategy shown.**


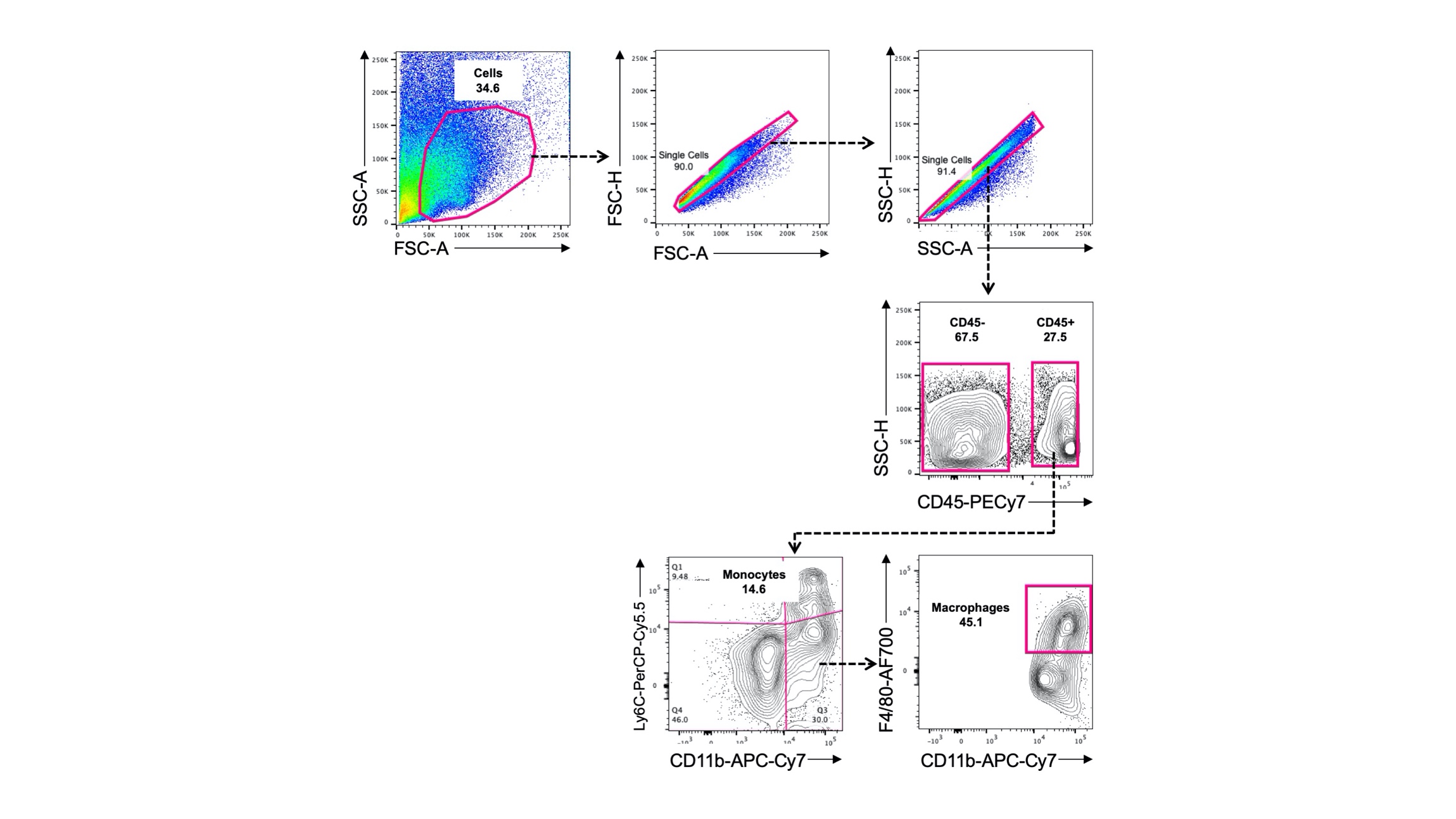

Supplement: Supplementary file 1 [file Data_Sheet_1.docx]
